# Optimal Weighting of Preclinical Alzheimer’s Cognitive Composite (PACC) Scales to Improve their Performance as Outcome Measures for Alzheimer’s Disease Clinical Trials

**DOI:** 10.6000/1929-6029.2023.12.12

**Published:** 2023-09-07

**Authors:** Xinran Wang, Diane Jacobs, David P. Salmon, Howard H. Feldman, Steven D. Edland

**Affiliations:** 1 Division of Biostatistics, Herbert Wertheim School of Public Health and Human Longevity Science, University of California San Diego, 9500 Gilman Dr., La Jolla, CA 92093, USA; 2 Department of Neurosciences, School of Medicine, University of California San Diego, 9500 Gilman Dr., La Jolla, CA 92093, USA; 3 Alzheimer’s Disease Cooperative Study, University of California San Diego, 9500 Gilman Dr., La Jolla, CA 92093, USA

**Keywords:** Cognitive composite, statistical power, clinical trial design, clinical trial endpoints

## Abstract

**Introduction:**

Cognitive composite scales constructed by combining existing neuropsychometric tests are seeing wide application as endpoints for clinical trials and cohort studies of Alzheimer’s disease (AD) predementia conditions. Preclinical Alzheimer’s Cognitive Composite (PACC) scales are composite scores calculated as the sum of the component test scores weighted by the reciprocal of their standard deviations at the baseline visit. Reciprocal standard deviation is an arbitrary weighting in this context, and may be an inefficient utilization of the data contained in the component measures. Mathematically derived optimal composite weighting is a promising alternative.

**Methods:**

Sample size projections using standard power calculation formulas were used to describe the relative performance of component measures and their composites when used as endpoints for clinical trials. Power calculations were informed by (n=1,333) amnestic mild cognitive impaired participants in the National Alzheimer’s Coordinating Center (NACC) Uniform Data Set.

**Results:**

A composite constructed using PACC reciprocal standard deviation weighting was both less sensitive to change than one of its component measures and less sensitive to change than its optimally weighted counterpart. In standard sample size calculations informed by NACC data, a clinical trial using the PACC weighting would require 38% more subjects than a composite calculated using optimal weighting.

**Discussion:**

These findings illustrate how reciprocal standard deviation weighting can result in inefficient cognitive composites, and underscore the importance of component weights to the performance of composite scales. In the future, optimal weighting parameters informed by accumulating clinical trial data may improve the efficiency of clinical trials in AD.

## INTRODUCTION

The Alzheimer’s Disease Cooperative Study (ADCS) Preclinical Alzheimer Cognitive Composite (PACC) is a neuropsychometric assessment tool constructed by combining scores from four well-validated objective tests of global cognition and verbal memory performance [1]. The PACC was developed for and was the planned primary outcome measure of the Anti-Amyloid Treatment in Asymptomatic Alzheimer’s study, a phase 3 randomized clinical trial of solanezumab to slow the progression of memory problems in those who are cognitively normal but have a PET scan indicating brain amyloid pathology [1]. As of March 2023, 28 clinical trials listed in clinicaltrials.gov report a version of the PACC as a primary or secondary cognitive outcome measure, making the PACC one of the favored outcome measures used in AD clinical trials in those at risk of but not yet diagnosed with dementia. While the PACC was calibrated to measure cognitive decline in normal participants with brain amyloidopathy, it has also been applied to cohort studies and clinical trials of older adults with other predementia conditions including amnestic mild cognitive impairment (aMCI) [2, 3]. There is now an evolving and active literature describing variants of the PACC, including PACCs with more than four component tests [4–7] and PACCs with different component tests than used in the original PACC [4–6, 8]. A five-item version of the PACC was the planned primary endpoint for the “SKYLINE” phase 3 trial of gantenerumab in cognitively normal persons with biomarker evidence of amyloid accumulation [9]. Hence there are now multiple derived versions of the PACC, and they are seeing broad application in both small and large cohort studies and clinical trials.

The originally described PACC [1] and all subsequently derived PACC instruments z-score norm the component scores on baseline data before summing the components to form their composite. This introduces two limitations to PACC weighted scores. First, dividing each component by its baseline standard deviation effectively re-weights the component scores in an arbitrary and often extremely inefficient way. A second limitation is that meta-analyses of accumulating trial data will not be possible with PACC weighted composites – the baseline standard deviations of component measures vary from study to study, and consequently z-score normed PACCs are non-comparable (different) scales study to study.

We have shown using computer simulations informed by clinical trial data that required sample size using PACC weighting can be up to twice that required using the same component scales with optimal weighting [10]. Here we investigate the performance of PACC weighting empirically, using data from the National Alzheimer’s Coordinating Center (NACC) database. This exercise is intended to provide a more heuristic demonstration of PACC performance. Findings are that a PACC weighted composite is less efficient than the optimal composite, and that PACCs can even underperform relative to individual component instruments contained within the PACC. The latter observation, especially, highlights the arbitrary and potentially inefficient nature of composites weighted by baseline standard deviations.

## METHODS

### Study Material

Following Donohue, *et al*., [1] we investigated the performance of cognitive composites using an approximation of the PACC constructed from publicly available datasets. We examined longitudinal patterns of decline in participants in the NACC database [11], restricting to participants with a baseline visit diagnosis of aMCI. The NACC database contains cumulative longitudinal data from participants enrolled in cohort studies at 41 NIH funded Alzheimer’s Disease Research Centers (ADRC). The NACC Uniform Data Set (UDS) was initiated in 2005 and contains annually collected clinical diagnostic data and psychometric test data from all ADRCs. NACC UDS data were accessed in January of 2021. The NACC baseline aMCI sample included 1,333 participants who were above 60 years old, with a diagnosis of aMCI at their initial UDS visit, and at least three complete annual follow-up evaluations after their first UDS visit.

### Component Tests of the Cognitive Composites

The original PACC [1] includes the following four test scores: 1) total recall from the Free and Cued Selective Reminding Test (FCSRT) (score range = 0–48 points); 2) delayed recall on the Logical Memory IIA (LM-IIA) sub-test from the Wechsler Memory Scale (score range = 0–25 points); 3) total correct responses on the Digit Symbol Substitution Test (DSST) from the Wechsler Adult Intelligence Scale–Revised (score range = 0–93 points); and 4) the Mini-Mental Status Exam (MMSE) total (score range = 0–30 points).

The full set of component tests included in the PACC is not present in the NACC UDS cognitive test batteries. The NACC dataset includes the LM-IIA, DSST, and MMSE, but does not include the FCSRT or an equivalent. Thus, a “PACC3” was constructed as a three-item composite that including the three available tests (LM-IIA, DSST, MMSE), but no FCSRT or equivalent.

### Statistical Methods

This analysis evaluates the relative efficiency of outcome measures for clinical trials using the mixed model repeated measures (MMRM) analysis plan. MMRM is the most commonly used statistical analysis plan for phase 3 AD clinical trials, and compares the mean change from baseline to the last visit in the treatment arm versus the placebo arm [12]. To simplify presentation, we assumed no loss to follow-up and no covariates, so that the MMRM analysis reduces to a standard two sample t-test comparing change in treatment to change in placebo. This assumption is justified because our focus is the relative efficiency of different outcome measures rather than actually powering a future trial, and relative efficiency is only indirectly and modestly affected by missing value patterns and covariate terms. Power calculations further assumed equal allocation to arms, a type 1 error rate of 5%, and equal standard deviation of change in treatment and placebo arms. Effect size to be powered was set to a 50% reduction in the mean change from baseline to year three. The 50% effect size is an arbitrary percentage -- relative efficiency is independent of effect size when effect size is expressed as a percentage reduction in change [13], meaning any percentage effect size results in the same relative efficiency of outcome measures. Power calculations are summarized by reporting the sample size required to achieve 80% power. All analyses were conducted using R version 4.1.0. Power calculations were performed using the R base package power.t.test function.

PACC3 scores were calculated by dividing each component score by its standard deviation at the baseline visit and summing the resulting values [1]. That is, PACC3 scores are a weighted sum of reciprocal standard deviation weighted component scales. (Formal z-score norming with subtraction of the means could also be performed but is redundant to the MMRM analysis. All Tables and results reported here are identical with or without subtracting out the mean at this stage.) We also calculated composite scores of the three component instruments using optimal weighting and simple sum weighting. Optimal weighting applies weights calculated from the covariance of change scores [10]. Simple sum weighting, also called the unweighted sum approach, weights each component test equally when calculating the composite score.

Optimal composite weighting was first proposed by Xiong *et al*. in the context of linear mixed effects models comparing fixed effect mean slope between groups [14]. Xiong *et al*. used least squares arguments to derive a candidate formula for optimal component weights that maximizes the sensitivity of the composite to longitudinal change. However, in computer simulations the Xiong *et al*. composite performed more poorly than the simple sum composite formed by adding unweighted component scores, and the authors concluded that further research is required [14]. In a formal derivation of optimal weights from the multivariate distribution of the component scores, Ard *et al*. [15] demonstrated that optimal weights are a function of both the parameters of the joint multivariate distribution of the component scores and the clinical trial design (i.e., the number and interval evaluations during the clinical trial). The optimal composite as defined by Ard *et al*. is the weighted sum of component measures that maximizes the mean to standard deviation ratio (MSDR) of the expected change in the outcome measure, thereby minimizing the sample size required to power a cohort study or clinical trial using the composite as an outcome measure. The Ard *et al*. algorithm generalizes readily to the MMRM analysis preferred by clinical trialists [12]. The formula for optimal weights for the MMRM analysis can be expressed in matrix notation as

$$\text{Optimal weights}=c*\Sigma^{-1}{\text{μ}}^{\prime }$$

given expected change μ and covariance of change Σ [13]. The *c* is an arbitrary constant – any non-zero value of *c* will produce equally optimal weights. We use the convention of standardizing *c* so that the weights sum in absolute value to 1. Optimal weights reflect the covariance of change of the component scales, and result in the linear combination of components that maximizes the expected change in the composite in units of the standard deviation of change of the composite within the placebo arm.

We also report the mean to standard deviation ratio (MSDR) of change scores for each component measure and each composite. Because the effect size in power calculation formulas is expressed in units of MSDR, the MSDR is a useful metric for comparing the performance of different outcome measures. The MSDR is also known as the signal-to-noise ratio of the outcome measure. Outcomes with larger MSDR have a higher power and require a smaller sample size, while outcomes with smaller MSDR have lower power and require a larger sample size.

Finally, we report descriptive summaries of the different weighting schemes expressed on a standard scaling. Optimal weights by design are standardized so that the weights sum in absolute value to one [10]. We likewise standardized the PACC and simple sum weights to sum to one to be able to more readily compare weighting schemes. This was done by multiplying the weights by a constant term equal to one over the sum of the component weights [10, 13]. Multiplying weights by a constant term in this manner shifts the mean and standard deviation of the outcome measure but has no effect on its performance in terms of statistical testing and power calculations [13, 15]. Standardizing the weights in this way serves to create weights that are on the same referent scale and comparable across weighting approaches.

## RESULTS

**Figure 1:**
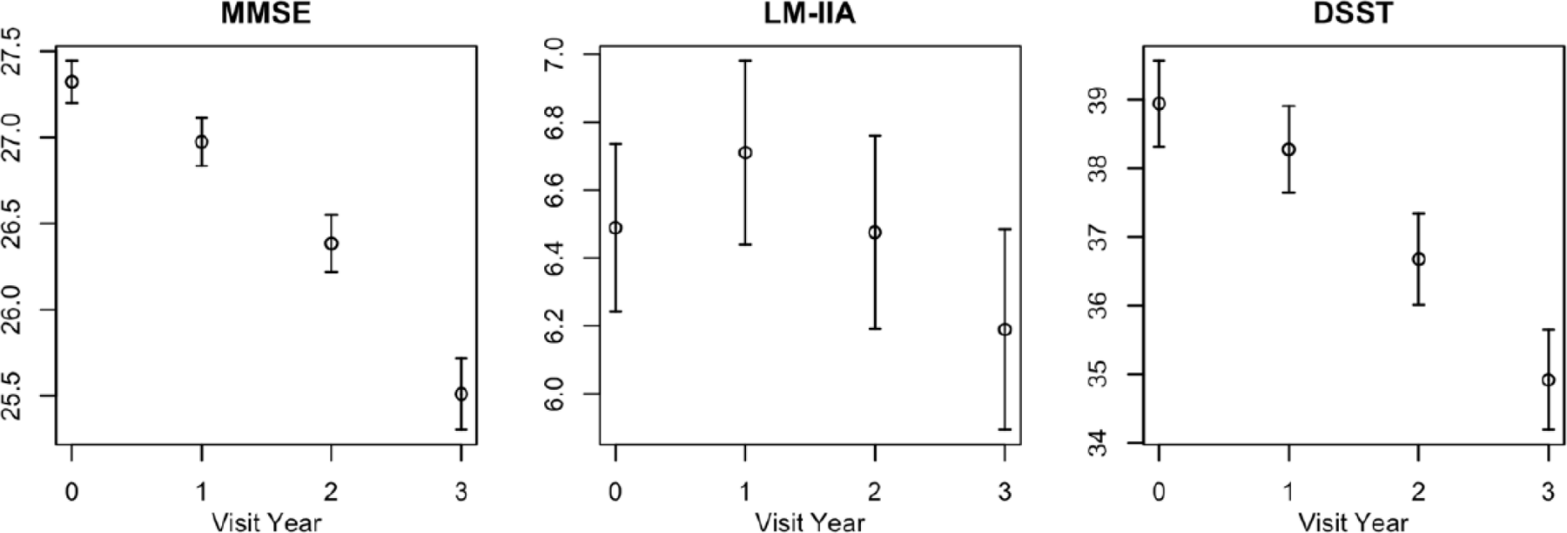
Mean (95% confidence intervals) for the component measures, by visit. MMSE = Mini-Mental State Exam; LM-IIA = Logical Memory Paragraph Recall; DSST = Digit Symbol Substitution Test.

Demographic characteristics of the NACC aMCI participants are summarized in Table 1. Mean age at enrollment was 74.8 years. Forty-six percent of the participants were women. Participants were predominantly non-Hispanic White (79.6%), and well educated (mean 15.4 years), as is typical of volunteer registry cohorts [16]. Mean scores on the component neuropsychometric instruments are also summarized on Table 1. Graphical summaries of the mean scores at baseline and the three annual follow-up evaluations are presented in Figure 1. Subjects declined on average on each of the PACC3 component measures, although there was a slight increase in mean score for the paragraph recall (LM-IIA) instrument from baseline to first follow-up visit (Figure 1). Mean change from first to last visit was highly statistically significant for the MMSE and DSST (p < 0.0001), and less so for the LM_IIA (p = 0.007).

Table 2 shows the mean change and standard deviation of change from baseline to year three for each component test and for composite measures formed from the component tests. Table 2 also reports the MSDR and the sample size required to observe a statistically significant 50% reduction in mean change in treatment versus placebo with 80% power. Based on data from NACC aMCI participants, a clinical trial using the PACC3 would require 246 participants per arm in this target population. The three-item PACC3 outperformed the three-item simple sum composite which would require 268 participants per arm, but not the optimally weighted three-item composite which would require 177 participants per arm (Table 2). Compared to the optimally weighted composite, the PACC3 would requires 38% more subjects for a comparably powered trial. We also observe that PACCs can underperform relative to individual component instruments contained within the PACC (Table 1, comparing MMSE alone (N=222/arm) to the PACC3 with PACC weighting (N=246/arm)). Stated another way, adding the LM-IIA and DSST to the MMSE using reciprocal standard deviation resulted in a loss of statistical power for the scale when used as an outcome for a clinical trial.

Weights for calculating the PACC3 scales are summarized in Table 3. Standardized weights for the PACC weighted and the simple sum and optimally weighted scales are also summarized in Table 3. The standardized weights (Table 3) in combination with the MSDRs (Table 2) help to explain the relative efficiency of the different scales. In general, component scales with small MSDR contribute little to the composite scale, and hence composite scales which give higher weight to component scales with small MSDR perform poorly. For example, the LM-IIA had the smallest MSDR, meaning it is a noisy instrument relative to the other component measures, but was substantially up weighted by the PACC3 relative to the optimal weighting.

## DISCUSSION

This study presents analyses of reciprocal standard deviation weighted composites as outcome measures for clinical trials, and compares different weighting approaches to scoring a PACC. We found a number of results relevant to the construction of cognitive composites. We illustrated by counter example that adding additional components to a composite does not necessarily improve the performance of the composite.

Our summary also revealed that the LM-IIA improved from baseline to first follow-up in the NACC aMCI data (Figure 1, panel 3), consistent with practice effects on this measure in the aMCI population. Composites using component tests with practice effects may benefit from a run in period to improve the signal-to-noise of the outcome measure and increase statistical power [17]. We also note that the LM-IIA is not sensitive to change in the population of aMCI participants represented in the NACC cohort, and has little power to detect treatment effects as a stand-alone outcome measure (Table 2).

A limitation of all attempts to project the statistical power of a planned clinical trial is that there are typically no *a priori* data to inform the likely pattern of progression under treatment. This is especially true for Alzheimer’s disease prevention and treatment trials, which have experienced a significant history of negative findings and therefore little data to inform the effect of treatment on trial outcome variables. The standard approach to powering trials in this circumstance is to use the covariance pattern of representative placebo arm data and assume this covariance pattern for both the placebo and treatment arms. This is the approach we have taken in this and previous [10, 15] papers. The equal covariance assumption may not hold in practice. For example, response to treatment under the alternative hypothesis will be variable, meaning the change from baseline to last visit in the treatment arm of a clinical trial will reflect both natural background variability in rate of progression and variability in response to treatment. Under this scenario the equal covariance assumption may lead to anticonservative sample size estimates. Conversely, an especially effective treatment may reduce the variance of change in the treatment arm. In the hypothetical extreme, a treatment that stopped progression would constrain the variance of change to be a function of only the measurement error variance of repeated measures. Under this scenario, the equal covariance assumption would lead to conservative sample size estimates. An alternative to the equal covariance assumption approach is to construct a comparator sample similar to what might be achieved under treatment [1]. This approach can lead to anticonservative power projections if the pattern of longitudinal change in the comparator group is not representative of what will be experienced in future clinical trials. We also note that the optimal weighting algorithm assumes that treatment effects expressed as a percentage slowing of decline are equivalent across the component measures [15]. Recent findings suggest that this may be a reasonable assumption. Published data on recently completed clinical trials of effective monoclonal antibody therapies for the treatment of early AD showed remarkably similar treatment effects by percentage change across the outcome measures used in the trials [18, 19]. For example, in the lecanemab monoclonal antibody trial, treatment slowed progression by comparable percentages over 18 months on both the primary global assessment outcome measure (27%), and on the planned secondary cognitive and functional outcome measures (26–37%) [18]. Assessing the assumptions of the optimal composite approach is critical. As more trials with positive effects read out, we will be able to more thoroughly investigate the many assumptions implicit in sample size and statistical power calculations by this approach.

Finally, we note that a feature of the optimal composite approach is that prior study data from a representative completed clinical trial or instrument protocol study is required to estimate the parameters used to calculate optimal weights [15]. Optimal weights estimated from the data to which the composite are applied, as reported here, may overstate the power of the composite in a future clinical trial [15, 20]. We have found that optimal weights estimated from pilot samples as small as 200 participants are effective approximations of weights that achieve theoretical maximal power [10]. The NACC sample reported here well exceeds this threshold, but we emphasize that the relative performance of the optimal weights may be slightly overstated in this analysis. In application, optimal weights should be from prior independent sample data representative of the planned future trial.

## CONCLUSIONS

We have demonstrated that composite scales with reciprocal standard deviation weighting can be an inefficient use of clinical trial data. A practical indication of this is that the single component MMSE measure was more efficient than the full PACC3. That is, adding more information in the form of more tests to a reciprocal standard deviation weighted composite can actually decrease the efficiency of the scale as an outcome measure for clinical trials. These observations provide useful guidance to future investigators developing composite outcome measures for cohort studies and clinical trials. Considering weighting when constructing composite scales may improve the statistical efficiency of clinical trials moving forward.

## Figures and Tables

**Table 1:** Baseline and Year 3 Characteristics of NACC aMCI Participants

(n = 1,333)	Baseline	Year 3
Entry Age, mean (SD*)	74.8 (7.3)	
Education, mean (SD)	15.4 (3.2)	
Female Sex, n (%)	613 (46.0%)	
Non-Hispanic White, n (%)	1061 (79.6%)	
Non-Hispanic Black, n (%)	162 (12.2%)	
Non-Hispanic Asian, n (%)	22 (1.7%)	
Hispanic White, n (%)	57 (4.3%)	
Other, n (%)	31 (2.3%)	
**PACC Components**		
MMSE, mean (SD)	27.3 (2.3)	25.5 (3.9)**
DSST, mean (SD)	38.9 (11.7)	34.9 (13.6)**
LM-IIA, mean (SD)	6.5 (4.6)	6.2 (5.5)***

* SD = standard deviation

** p<0.00001

*** p=0.007

**Table 2:** Summary Statistics and Sample Size Estimates for Change Baseline to Year Three

	Mean Change (SD)	MSDR*	Sample Size**
PACC3 components			
MMSE	−1.81 (3.40)	−0.53	222
DSST	−4.03 (8.99)	−0.45	314
LM-IIA	−0.30 (4.03)	−0.07	11390
PACC3 composite scales			
PACC weighting	−1.20 (2.38)	−0.51	246
Optimal weighting	−1.84 (3.08)	−0.60	177
Simple sum weighting	−6.14 (12.67)	−0.48	268

* MSDR = mean to standard deviation ratio

** Sample size per arm required to detect a 50% slowing of decline under treatment with 80% power.

**Table 3:** PACC3 Component Weights and Standardized Component Weights vis-à-vis Optimal and Simple Sum Weights

	MMSE	LM-IIA	DSST
Baseline SD*	2.28	4.60	11.68
PACC weights (= 1/SD)	0.44	0.22	0.09
Standardized weights			
PACC	0.59	0.29	0.12
Optimal composite	0.68	−0.16	0.16
Simple sum composite	0.33	0.33	0.33

* SD = standard deviation.
